# Supplementary material for: Sleep bolsters schematically incongruent memories
Source: PLoS One. 2022 Jun 24;17(6):e0269439. doi: 10.1371/journal.pone.0269439 (PMC9231735; doi:10.1371/journal.pone.0269439)
Supplement: S1 File — (DOCX) [file pone.0269439.s001.docx]

**Supplementary Results**

In a subsidiary analysis, we assessed whether the sleep vs wake group difference in item memory at the 12 h follow-up could account for the between-group difference in source memory performance. To this end, we repeated our 2 (Group: Sleep/Wake) * 3 (Rehearsal Strategy: Restudy/Retrieval Practice/No-Rehearsal) * 2 (Plausibility: Plausible/Implausible) mixed ANOVA on source memory scores (colour hits) at the 12 h follow-up, but fully controlled for item memory performance by restricting the input data to colours for which the associated noun was correctly recognised at both the baseline test and the 12 h follow-up. The relevant data is shown in Supplementary Table 1.

Confirming the results of our main analysis, there was a main effect of Rehearsal Strategy (*F*(2, 116) = 37.12, *p* < .001, *η_p_^2^* = 0.39): retention scores were higher in 1) the restudy vs no-rehearsal condition (*t*(59) = 8.45, *p* < .001, *d* = 1.09), 2) the retrieval practice vs no-rehearsal condition (*t*(59) = 5.70, *p* < .001, *d* = 0.74), and 3) the restudy vs retrieval practice condition (*t*(58) = 2.75, *p* = .007, *d*= 0.36), indicating that restudy produced the greatest overall retention benefit. There was also a main effect of Plausibility (*F*(1, 58) = 73.90, *p* < .001, *η_p_^2^* = 0.56), indicating that source memory retention was higher for plausible relative to implausible noun-colour pairings, and a main effect of Group (*F*(1, 58) = 10.54, *p*  = .002, *η_p_^2^* = 0.15), indicating that source memory retention was higher after a night of sleep than a day of wakefulness.

There was also a Group * Plausibility interaction (*F*(1, 58) = 6.02, *p* = .017, *η_p_^2^* = 0.09). Again, post-hoc comparisons revealed that the retention advantage for plausible (vs implausible) noun-colour pairings was smaller in the sleep group (*t*(29) = 5.04, *p* < .001, *d* = 0.92) than the wake group (*t*(29) = 6.97, *p* < .001, *d* = 1.27), and the memory benefits of sleep (vs wakefulness) were greater for implausible pairings (*t*(58) = 3.24, *p* = .002, *d* = 0.84) than plausible pairings (*t*(58) = 2.68, *p* = .010, *d* = 0.69). Taken together, these findings suggest that sleep preferentially facilitates the consolidation of implausible (and thus schematically incongruent) associations. All other effects were non-significant (all *p* > .05).

We also repeated our analysis of source memory retention at the 24 h follow-up, restricting this to colours for which the associated noun was correctly recognised at both the baseline test and the 24 h follow-up. Confirming the results of our main analysis, there was a main effect of Plausibility (*F*(1,58) = 51.61, *p* < .001, *η_p_^2^* = 0.47) and a main effect of Rehearsal Strategy (*F*(2,58) = 32.74, *p* < .001, *η_p_^2^* = 0.36). However, the Group * Rehearsal interaction was a non-significant trend (*F*(2,116) = 2.47, *p* = .089, *η_p_^2^* = 0.04). All other effects were non-significant (all *p* > .05).

|  | **Restudy** | | **Retrieval Practice** | | **No-Rehearsal** | |
| --- | --- | --- | --- | --- | --- | --- |
|  | **Plausible** | **Implausible** | **Plausible** | **Implausible** | **Plausible** | **Implausible** |
| ***12 h Follow-up*** | | | | | | |
| Sleep | 91.58 ± 1.29 | 87.28 ± 2.50 | 87.59 ± 1.41 | 78.78 ± 2.26 | 82.18 ± 1.86 | 73.96 ± 2.41 |
| Wake | 85.81 ± 2.45 | 74.94 ± 4.03 | 83.61 ± 2.06 | 74.05 ± 3.35 | 76.74 ± 1.75 | 58.82 ± 3.45 |
| ***24 h Follow-up*** | | | | | | |
| Sleep | 86.74 ± 2.00 | 83.66 ± 2.43 | 83.50 ± 2.30 | 73.17 ± 2.60 | 78.99 ± 2.38 | 69.65 ± 2.82 |
| Wake | 85.66 ± 1.87 | 76.85 ± 2.91 | 83.60 ± 2.28 | 74.98 ± 2.79 | 76.54 ± 2.91 | 62.29 ± 2.99 |

**S1 Table.** Source memory scores (%) for the 12 h and 24 h follow-ups in the sleep and wake groups, restricted to colours for which the associated noun was correctly recognised at both the baseline test and the 12 h follow-up (top rows) or the 24 h follow-up (bottom rows). Scores are presented separately for each rehearsal strategy and plausibility condition. Data are presented as mean ± SEM.
