# Supplementary material for: Sleep bolsters schematically incongruent memories
Source: PLoS One. 2022 Jun 24;17(6):e0269439. doi: 10.1371/journal.pone.0269439 (PMC9231735; doi:10.1371/journal.pone.0269439)
Supplement: Attachment — Submitted filename: PLOS_R1_ReviewerResponse_Final.docx [file pone.0269439.s002.docx]

**Reviewer #1**

In this manuscript, Ashton and colleagues investigate the role of rehearsal strategy, schema congruency and sleep on cued recall of colors previously associated with nouns. In an online study, two groups of participants learned noun-color pairs by deciding whether the pairing was plausible or not either in the morning or in the evening. Their memory was tested at 12h and 24h delays, showing the expected main effects at the 12h interval. At 24h, the difference between restudy and retrieval remained only for the sleep group, which according to the authors indicates a critical time window shortly after learning for sleep-related benefits on restudied information. Furthermore, a significant interaction revealed group differences between memory for plausible and implausible items. The manuscript is concise and well written; however, I see several issues, especially concerning interpretation of the results.

24h sleep*rehearsal interaction:

1) First, according to the description, participants were tested on all learned stimuli at 12h. Why did the authors choose to do so? This is an unnecessary complication, because this means that at 24h the items in the ‘restudy’ condition actually underwent restudy and retrieval practice. It should be taken into account that under these circumstances especially the emerging differences between restudy and retrieval practice conditions at 24h is hard to interpret.

**Authors:** We thank the Reviewer for this comment. We chose to test all learned noun-colour pairings at both the 12 h and 24 h follow-ups to ensure that there would not be a paucity of stimuli in the various rehearsal conditions, which would have reduced our statistical power.

To elaborate, under our current approach (taking the noun-colour pairings that were correctly retrieved at baseline and dividing these equally between the restudy, retrieval practice and no-rehearsal conditions), we had a mean of 41.81 (± 1.29 SEM) pairings per condition (all of which were assessed at both the 12 h and 24 h follow-ups). As an alternative, we could have assessed retention for half of the noun-colour pairings at the 12 h follow-up and the other half at the 24 h follow-up, but we would have had only 21 pairings per condition, and these would have been further subdivided by the plausibility condition (i.e. whether participants considered the noun-colour pairings to be plausible or implausible at learning).

When weighing up this trade-off during the design phase of our study, we chose to assess all items at both the 12 h and 24 h follow-ups because this approach ensured that our experiment would be sufficiently powered and, resultantly, provide a concrete assessment of the effects of sleep, rehearsal and schematic congruency on source memory.

We nevertheless agree with the Reviewer that our chosen approach introduces a degree of complexity into the interpretation of our findings at the 24 h follow-up. We have now added new text to our Discussion, where we acknowledge this concern and explain the logic behind our methodological decisions:

Page 17: *A further point to consider is that participants’ memory for all noun-colour pairings was assessed at both the 12 h and 24 h follow-ups, which meant that pairings in the restudy condition had undergone both restudy and retrieval practice by the time of the 24 h assessment. We therefore cannot rule out the possibility that the selective influence of sleep on restudied information at the 24 h follow-up was influenced by retrieval at the 12 h follow-up. We chose to test all noun-colour pairings at both follow-ups to ensure that there would be a sufficient number of stimuli for the various rehearsal conditions. For example, despite participants encoding 240 noun-colour pairings, only 125.43 (mean ± SEM, ± 3.87) of these were correctly retrieved at baseline, meaning that each rehearsal condition contained 41.81 (± 0.29) pairings. As an alternative, we could have assessed retention for half of the noun-colour pairings at the 12 h follow-up and the other half at the 24 h follow-up, but this would have left only ~21 pairings per rehearsal condition, which would have been further subdivided by the plausibility condition. We therefore believe that our chosen approach was optimal, given the various research questions that we sought to address. Future work focusing on the relationship between overnight consolidation and rehearsal (independent of schematic congruency) can build on our preliminary findings by assessing the isolated effects of restudy and retrieval practice on sleep-associated memory processing.*

2) Second, interpreting a difference between the two time points (“time-critical relationship between memory rehearsal and sleep-associated memory gains”) should be secured with an appropriate statistical test including both time points.

**Authors:** We acknowledge that our interpretation of a “time-critical relationship between memory rehearsal and sleep-associated memory gains” was too strong, given the nature of our findings. We have therefore toned-down this claim in the relevant section of our manuscript:

Page 16: *Previous studies have demonstrated that overnight consolidation effects are strongest for information learned within a few hours of sleep (Ashton & Cairney, 2021; Gais, Lucas, & Born, 2006; Payne, et al., 2012; Talamini et al., 2008). Our findings build on this prior work by suggesting that the selective benefits of sleep for restudied information are contingent on sleep taking place soon after learning.*

In light of this change, we believe that further statistical analysis (beyond that outlined in our pre-registration) is not warranted in this instance.

3) According to the methods section, participants were asked to recall the corresponding color association only if they judged a noun as old, regardless of whether it actually was an old or new item. Therefore the employed measure of memory performance is not only dependent on the ability to recall the color association in relation to the baseline test, but also on the ability to correctly recognize the item. Are there differences between the different groups/conditions in item memory that could have led to the observed differences in ‘source’ memory performance? Please include into the manuscript.

**Authors:** We are very grateful to the Reviewer for raising this important point. Although our analyses of source (colour) memory were restricted to colours for which the associated noun was correctly recognised at the baseline test, we did not control for item memory (noun recognition) at the 12 h or 24 h follow-ups. As correctly pointed out by the Reviewer, between-condition differences in item memory at the follow-up tests could be misinterpreted as a difference in source memory. We have taken multiple steps to address this concern, as outlined below.

First, we looked at whether item memory (noun recognition) at the 12 h follow-up differed between the sleep and wake groups. We calculated d-prime to determine recognition accuracy for both groups and entered these scores into an independent samples t-test. Importantly, item memory at the 12 h follow-up was significantly better in the sleep group than the wake group (t(58) = 2.01, p = .049). Please note that we were unable to obtain a separate measure of d-prime for each of our within-subjects conditions (Rehearsal Strategy: Restudy/Retrieval Practice/No-rehearsal and Plausibility: Plausible/Implausible) because this would require that each condition had its own independent set of foils.

Next, we assessed whether this between-group difference in item memory at the 12 h follow-up could explain the apparent between-group difference in source memory. To this end, we repeated our 2 (Group: Sleep/Wake) * 3 (Rehearsal Strategy: Restudy/Retrieval Practice/No-Rehearsal) * 2 (Plausibility: Plausible/Implausible) mixed ANOVA on source memory scores (colour hits) at the 12 h follow-up, but fully controlled for item memory performance by restricting the analysis to colours for which the associated noun was correctly recognised at both the baseline test and the 12 h follow-up (our main analysis only controls for item memory at the baseline test).

Crucially, we replicated the main effect of sleep observed in our main analysis (F(1, 58) = 10.54, p = .002, η_p_^2^ = 0.15), demonstrating that source memory was better after sleep than wakefulness at the 12 h follow-up, irrespective of item memory performance. The main effects of Rehearsal Strategy (F(2, 116) = 37.12, p < .001, η_p_^2^ = 0.39) and Plausibility were also replicated (F(1, 58) = 73.90, p < .001, η_p_^2^ = 0.56), and all other (non-significant) effects remained non-significant (see details below).

We also repeated our analysis of source memory performance at the 24 h follow-up, restricting this to colours for which the associated noun was correctly recognised at both the baseline test and the 24 h follow-up. The effects observed in our original analysis remained the same (see details below), except for the Group*Rehearsal interaction, which became a non-significant trend (F(2,116) = 2.47, p = .089, η_p_^2^ = 0.04). There was no difference in item memory between the sleep and wake groups at the 24 h follow-up (t(58) = 0.26, p = 0.80).

In sum, we have demonstrated that the results of our source memory analysis are (in the main) not influenced by the effects of sleep on item memory. Because other readers might raise similar concerns to the Reviewer, we have made the following changes to our manuscript.

1) We have added details of our item memory analysis to the Methods and Results sections:

Methods - Page 8: *D-prime [normalised (hits / hits + misses) – normalised (false alarms / false alarms + correct rejections)] was calculated to assess recognition accuracy for nouns (item memory) at the baseline test and the 12/24 h follow-ups. Independent samples t-tests comparing d-prime between the sleep and wake groups were performed at each test.*

Results - Page 9: *There was no difference in item memory (noun recognition accuracy, d-prime) between the sleep and wake groups at the baseline test (sleep group mean ± SEM: 2.09 ± 0.07, wake group: 2.11 ± 0.10, t(58) = 0.21, p = .839). However, at the 12 h follow-up, item memory was higher for participants who had slept (2.00 ± 0.10) relative to those who had remained awake (1.71 ± 0.11, t(58) = 2.01, p = .049, d = 0.52). No between-group difference was present at the 24 h follow-up (sleep group: 2.01 ± 0.10, wake group: 1.97 ± 0.13, t(58) = 0.26, p = 0.80).*

2) We have added a new Supplementary Results document to our paper, which provides a full breakdown of our subsidiary analyses of the 12 h and 24 h follow-ups. Because our original analyses were pre-registered, we have not replaced these with our new subsidiary analyses in the main manuscript. The main text of this supplementary section is copied below.

Supplementary Results: *In a subsidiary analysis, we assessed whether the sleep vs wake group difference in item memory at the 12 h follow-up could account for the between-group difference in source memory performance. To this end, we repeated our 2 (Group: Sleep/Wake) * 3 (Rehearsal Strategy: Restudy/Retrieval Practice/No-Rehearsal) * 2 (Plausibility: Plausible/Implausible) mixed ANOVA on source memory scores (colour hits) at the 12 h follow-up, but fully controlled for item memory performance by restricting the input data to colours for which the associated noun was correctly recognised at both the baseline test and the 12 h follow-up. The relevant data is shown in Supplementary Table 1.*

*Confirming the results of our main analysis, there was a main effect of Rehearsal Strategy (F(2, 116) = 37.12, p < .001, η_p_^2^ = 0.39): retention scores were higher in 1) the restudy vs no-rehearsal condition (t(59) = 8.45, p < .001, d = 1.09), 2) the retrieval practice vs no-rehearsal condition (t(59) = 5.70, p < .001, d = 0.74), and 3) the restudy vs retrieval practice condition (t(58) = 2.75, p = .007, d= 0.36), indicating that restudy produced the greatest overall retention benefit. There was also a main effect of Plausibility (F(1, 58) = 73.90, p < .001, η_p_^2^ = 0.56), indicating that source memory retention was higher for plausible relative to implausible noun-colour pairings, and a main effect of Group (F(1, 58) = 10.54, p = .002, η_p_^2^ = 0.15), indicating that source memory retention was higher after a night of sleep than a day of wakefulness.*

*There was also a Group * Plausibility interaction (F(1, 58) = 6.02, p = .017, η_p_^2^ = 0.09). Again, post-hoc comparisons revealed that the retention advantage for plausible (vs implausible) noun-colour pairings was smaller in the sleep group (t(29) = 5.04, p < .001, d = 0.92) than the wake group (t(29) = 6.97, p < .001, d = 1.27), and the memory benefits of sleep (vs wakefulness) were greater for implausible pairings (t(58) = 3.24, p = .002, d = 0.84) than plausible pairings (t(58) = 2.68, p = .010, d = 0.69). Taken together, these findings suggest that sleep preferentially facilitates the consolidation of implausible (and thus schematically incongruent) associations. All other effects were non-significant (all p > .05).*

*We also repeated our analysis of source memory retention at the 24 h follow-up, restricting this to colours for which the associated noun was correctly recognised at both the baseline test and the 24 h follow-up. Confirming the results of our main analysis, there was a main effect of Plausibility (F(1,58) = 51.61, p < .001, η_p_^2^ = 0.47) and a main effect of Rehearsal Strategy (F(2,58) = 32.74, p < .001, η_p_^2^ = 0.36). However, the Group * Rehearsal interaction was a non-significant trend (F(2,116) = 2.47, p = .089, η_p_^2^ = 0.04). All other effects were non-significant (all p > .05).*

3) We have explicitly mentioned this subsidiary analysis in our main manuscript text:

Page 11: *Although the foregoing analyses of source memory retention were restricted to colours for which the associated noun was correctly recognised at baseline, they did not control for noun recognition (item memory) at the 12 h or 24 h follow-ups. This is an important consideration, because if a participant failed to re-recognise a noun at the 12 h follow-up, the trial was scored as a source memory error, meaning that any effect of sleep on item memory could have been misinterpreted as an effect of sleep on source memory. To address this concern, we repeated the above analyses (and those described in the next section), but fully controlled for item memory by restricting the input data to colours for which the associated noun was correctly recognised at both the baseline test and the relevant 12 h or 24 h follow-up (see Supplementary Results). In brief, these subsidiary analyses confirmed the results of our main analyses, although the Group * Rehearsal interaction at the 24 h follow-up became a non-significant trend (F(2,116) = 2.47, p = .089, η_p_^2^ = 0.04).*

4) We have explicitly acknowledged the non-significant Group * Rehearsal interaction from our reanalysis of the 24 h follow-up in our Discussion:

Page 16: *Although rehearsal strategy had no effect on the memory benefits of sleep at the 12 h follow-up, a significant interaction emerged at the 24 h follow-up, such that restudied memories were better remembered than retested memories, but only in the sleep group (i.e. those individuals who had slept soon after learning). However, this interaction became a non-significant trend in a subsidiary analysis that controlled for item memory performance at the baseline test and 24 h follow-up.*

4) From my point of view, the conclusion that sleep preferentially strengthens implausible memories is a bit misleading. It is a pity that there is no data on performance right after the rehearsal manipulation to get a better look at the isolated effect of sleep on schema-congruency, however when comparing baseline and 12h performance in the no rehearsal condition, it seems more that sleep stabilizes plausible and implausible items alike (same performance levels) and thereby counteracts stronger forgetting of implausible information seen in the wake group.

**Authors:** We agree with the Reviewer that our data indicate a general benefit of sleep on the retention of both plausible and implausible memories at the 12 h follow-up (as indicated by the main effect of Group in our ANOVA and illustrated in Figure 4A). Importantly, however, Figure 4A also clearly shows that the difference in retention for plausible and implausible memories at the 12 h follow-up is reduced after sleep relative to wakefulness (corresponding to a significant Group * Plausibility interaction).

One interpretation of this finding is that the benefits of sleep for memory are amplified for schematically incongruent (i.e. implausible) relative to schematically congruent (i.e. implausible) associations. If this interpretation is correct, then the memory benefits of sleep (vs wakefulness) should be stronger for implausible pairings than plausible pairings (as suggested by Reviewer 2, comment 4). Bonferroni-corrected post-hoc tests revealed that this was the case. Our Results section has been updated to include these comparisons:

Page 12: *Post-hoc comparisons revealed that the retention advantage for plausible (vs implausible) pairings was smaller in the sleep group (t(29) = 6.18, p < .001, d = 1.13) than the wake group (t(29) = 8.56, p < .001, d = 1.56, see Figure 4A). Moreover, the memory benefits of sleep (vs wakefulness) were stronger for implausible pairings (t(58) = 3.41, p = .001, d = .88) than plausible pairings (t(58) = 3.00, p = .004, d = .78). Taken together, these findings suggest that sleep preferentially facilitates the consolidation of implausible (and schematically incongruent) associations.*

5) Why do the authors call a cued recall of associated color information ‘source memory’?

**Authors:** We refer to colour recall as source memory because the colour associated with each noun reflects the context or source with which that noun was encountered at learning. We have added the following text to our Methods to make this clear:

Page 7: *Note that we refer to colour recall as source memory because the colour associated with each noun reflects the context or source with which that noun was encountered at learning.*

6) How many items were judged as plausible in total and how were they distributed to the different conditions? Please report.

**Authors:** We thank the Reviewer for highlighting this omission. Across both the sleep and wake groups, participants rated 57% (SEM ± 1.41) of the noun-colour pairings as plausible. We have now added this information to the Methods section:

Page 7: *Across both the sleep and wake groups, participants rated 57% (SEM ± 1.41) of the noun-colour pairings as plausible.*

To check whether there was any bias in the distribution of noun-colour pairings between conditions, we applied colour hits at the baseline test to a 2 (Plausibility: Plausible/Implausible) * 3 (Rehearsal Strategy: Restudy/Retrieval Practice/No-Rehearsal) * 2 (Group: Sleep/Wake) mixed ANOVA. There was a main effect of Plausibility (F(1, 58) = 21.73, p < .001, η_p_^2^ = 0.27), reflecting participants’ tendency to rate more noun-colour pairings as plausible than implausible at encoding. There was also a main effect of Rehearsal Strategy (F(2, 116) = 3.13, p = .048, η_p_^2^ = 0.05), reflecting a slight difference in the number of pairings allocated to each rehearsal condition (mean ± SEM, Restudy: 21.08 ± 0.64, Retrieval Practice: 20.82 ± 0.65, No-Rehearsal: 20.83 ± 0.66). There was no main effect of Group and none of the interactions were significant (all p > .05).

The main effect of Rehearsal Strategy emerged because of a randomisation error, where on occasions that the number of baseline colour hits was not divisible by 3, the first ‘additional’ noun-colour pairing was always allocated to the restudy condition. For example, if 61 noun-colour pairings were correctly recalled at baseline, then 20 would be allocated to retrieval practice, 20 would be allocated to no-rehearsal and the remaining 21 would be allocated to restudy. We were not previously aware of this error and are very grateful to the Reviewer as their comment brought it to our attention.

Because our analyses of the 12 h and 24 h follow-ups refer to the proportion of noun-colour pairings that were retained from the baseline test, we do not think that this randomisation error poses any major problems for our results and conclusions. We have nevertheless, updated our Methods section to include details of this error:

Page 8: *To check whether there was any bias in the distribution of noun-colour pairings between conditions, we applied colour hits at the baseline test to a 2 (Plausibility: Plausible/Implausible) * 3 (Rehearsal Strategy: Restudy/Retrieval Practice/No-Rehearsal) * 2 (Group: Sleep/Wake) mixed ANOVA. There was a main effect of Plausibility (F(1, 58) = 21.73, p < .001, η_p_^2^ = 0.27), reflecting participants’ tendency to rate more noun-colour pairings as plausible than implausible at encoding. There was also a main effect of Rehearsal Strategy (F(2,116) = 3.13, p = .048, η_p_^2^ = 0.05 ), reflecting a slight difference in the number of pairings allocated to each rehearsal condition (mean ± SEM, Restudy: 21.08 ± 0.64, Retrieval Practice: 20.82 ± 0.65, No-Rehearsal: 20.83 ± 0.66). There was no main effect of Group and none of the interactions were significant (p > .05).*

*The main effect of Rehearsal Strategy emerged because of a randomisation error, where on occasions that the number of baseline colour hits was not divisible by 3, the first ‘additional’ noun-colour pairing was always allocated to the Restudy condition. For example, if 61 noun-colour pairings were correctly recalled at baseline, then 20 would be allocated to Retrieval Practice, 20 would be allocated to No-Rehearsal and the remaining 21 would be allocated to Restudy.*

**Reviewer #2**

This is an interesting experiment that both replicates and seeks to extend prior work. It tackles timely questions and provides to the greater landscape of the sleep and memory research. The design of this study is clever and the manuscript is well written. However, there were some points that require clarification.

1) How many exposures were used in the retrieval and restudy sessions – is it just 1 additional restudy of all images/1 additional retrieval test? What did subjects do during the no-rehearsal control session? Please provide greater detail about these methods. Likewise, were participants asked if they actively tried to rehearse at all during the 12 hour intervals?

**Authors:** We are very grateful to the Reviewer for highlighting areas of our manuscript where further detail is required. In response to the Reviewer’s questions:

1) Participants received one additional exposure (restudy) or test (retrieval practice) for the noun-colour pairings allocated to each restudy/retrieval practice condition.
2) The no-rehearsal control does not refer to a session but rather to a control condition. Of the noun-colour pairings that were correctly recalled at the baseline test, one third were allocated to the restudy condition, another third to the retrieval practice condition and the final third to the no-rehearsal control condition (wherein the noun-colour pairings were not revisited until the 12 h follow-up test). Hence, at the 12 h follow-up, it was possible to compare retention of noun-colour pairings that were restudied, had undergone retrieval practice or had not undergone any rehearsal.
3) Participants were not asked if they had undertaken any active rehearsal of the noun-colour pairings during the intervals between experimental sessions.

All of the above points have now been integrated into the relevant sections of the manuscript:

Page 7: *Participants received only one additional exposure to each noun-colour pairing allocated to the restudy and retrieval practice conditions. Pairings in the no-rehearsal condition were not revisited until the follow-up tests.*

Page 8: *Participants were not asked if they had undertaken any active rehearsal of the noun-colour pairings during the intervals between experimental sessions.*

2) Is the feedback on the baseline test only an overall score or are participants informed of which trials they got wrong? It seems possible that this feedback session could contribute to the restudy advantage, providing increased motivation to re-encode the pairs they have forgotten during the restudy. Could this potentially explain why immediately there is a restudy > retrieval effect, despite no interaction with sleep group?

**Authors:** Participants received feedback as an overall score and only at the end of the baseline test. As such, there was no indication to participants as to which noun-colour pairings were scored as correct or incorrect, and thus motivational factors were unlikely to account for the improved performance in the restudy (vs retrieval practice) condition at the follow-up tests. We have updated our Methods to reflect this important detail:

Page 7: *To motivate engagement with the task, participants were provided with an overall performance score at the end of the baseline test (calculated as the percentage of correct old/new responses) and were encouraged to try and improve on their score in the next test.*

3) How does source memory performance change after the retrieval rehearsal session? If the interpretation of the restudy vs retrieval benefit is that retrieval facilitates the rapid consolidation of information compared to restudy, it might provide nice additional support to test if there was a gain from baseline to post-retrieval, and perhaps not between post-retrieval to the 12 hour delay test.

**Authors:** The Reviewer has made a very interesting suggestion on how we might substantiate the claim that retrieval facilitates the rapid consolidation of newly formed memories. Unfortunately, however, our paradigm did not include a test immediately after the rehearsal phase, meaning that we cannot carry out the subsidiary analysis that the Reviewer suggests. Participants were only tested on three occasions: at baseline, at the 12 h follow-up and at the 24 h follow-up.

Only noun-colour pairings that were correctly recalled at the baseline test were allocated to the three rehearsal conditions, meaning that performance at the retrieval practice phase would have been at or near ceiling (because the rehearsal manipulation took place immediately after the baseline test).

4) For the 12 hour Group * Plausibility interaction: It would be informative to test if there is a significant difference between the implausible pairs, but not the plausible pairs, across the Sleep and Wake groups. If so, it seems like that comparison would provide better support for the idea that sleep, and not wake, specifically benefits the implausible pairs, whereas focusing on the reduced difference between the pair types says less about which items actually benefit.

**Authors:** We are very grateful to the Reviewer for this excellent suggestion. Bonferroni-corrected post-hoc comparisons revealed that the memory benefits of sleep (vs wakefulness) were stronger for implausible pairings than plausible pairings. Our Results section has been updated to include these new comparisons:

Page 12: *Post-hoc comparisons revealed that the retention advantage for plausible (vs implausible) pairings was smaller in the sleep group (t(29) = 6.18, p < .001, d = 1.13) than the wake group (t(29) = 8.56, p < .001, d = 1.56, see Figure 4A). Moreover, the memory benefits of sleep (vs wakefulness) were stronger for implausible pairings (t(58) = 3.41, p = .001, d = .88) than plausible pairings (t(58) = 3.00, p = .004, d = .78). Taken together, these findings suggest that sleep preferentially facilitates the consolidation of implausible (and schematically incongruent) associations.*

5) Were there any effects of Rehearsal Strategy using the exploratory ANOVA with the recalculated 24 vs 12 hr retention? It seems with the emergence of the Group x Rehearsal interaction only at 24 hours, a more direct comparison across the two delays would be helpful for understanding if this is an effect that only emerges over an extended period of time.

**Authors:** There was no main effect of Rehearsal Strategy (F(2,116) = 0.14, p = 0.87), no Rehearsal Strategy * Group interaction (F(2,116) = 0.69, p = .505) and no Rehearsal Strategy * Plausibility interaction (F(2,116) = 1.16, p = .318) in our ANOVA of the 24 h follow-up (recalculated on the 12 h follow-up). Because this was an exploratory analysis of the relationship between sleep and plausibility, we have only summarised these supplementary null effects in the main text:

Page 14: *All other effects in this exploratory analysis were non-significant (p > .05).*

6) On page 11, the authors state that the retention performance was higher for implausible than plausible associations in the wake group. Since this is from the exploratory data analysis using the 24/12hr retention measure, it is difficult to unpack this pattern from Figure 4, which seems to show a consistent plausible > implausible pattern at both time points and seemingly gains in both across the delays. Consider adding another Figure plotting the data from the exploratory analysis.

**Authors:** We thank the Reviewer for another excellent point, which has highlighted a labelling error in Figure 4B of our previous submission. Figure 4B was intended to illustrate the findings from our exploratory analysis on the 24 h follow-up (normalised on the 12 h follow-up); namely, the finding that performance was higher for implausible than plausible associations in the wake group. However, the plausible and implausible conditions were mislabelled, causing the problems outlined by the Reviewer in their comment.

To improve the clarity of our figures, we have now changed the content of Figure 4B so that it illustrates our main analysis of the 24 h follow-up (normalised on the baseline test, see Page 13). As pointed out by the Reviewer, this is easier to unpack alongside Figure 4A, which illustrates our analysis of the 12 h follow-up (also normalised on the baseline test).

What was previously Figure 4B (exploratory analysis of the 24 h follow-up, normalised on the 12 h follow-up) has now been moved to a new Figure 5 (see Page 14), wherein the labelling error from our previous manuscript has been corrected. Importantly, the pattern of higher retention performance for implausible vs plausible noun-colour pairings in the wake group is now clearly visible in this new Figure 5. We apologise for our labelling error and hope that these changes resolve the problems outlined in the Reviewer’s comment.

7) Likewise, for the exploratory analysis: the authors could provide greater discussion reconciling the non-significant Group main effect just at 24 hours with the significant Group interaction in this exploratory analysis (Wake > Sleep). Is this due to gains in the Wake group over the two sessions or forgetting in the sleep group? This seems central to the claims that the Wake group is getting a boost from the intervening sleep period.

**Authors:** We have added new text to the manuscript where we address this important point:

Page 14: *It is worth noting that the wake group even showed gains in retrieval performance between the 12 h and 24 h follow-ups (i.e. retention scores > 100%), which were especially pronounced for implausible noun-colour pairings, whereas the sleep group showed forgetting across both plausible and implausible pairings (thus explaining the non-significant Group main effect in our main analysis of the 24 h follow-up).*

8) Greater discussion attempting to reconcile the two sets of findings and what the results tell us more broadly about when and what sleep benefits is needed. Although it’s understandable that the interaction between schema consistency and rehearsal strategy was not statistically significant, there is still room to integrate more side by side discussion of the findings. For example, why might the benefit of sleep on restudied items be time-limited but not the plausibility effects? And similarly, why might the schema effects not be susceptible to the same potential ceiling effects at the 12 hour delay?

**Authors:** We are grateful to the Reviewer for this suggestion. We have updated our Discussion to provide a more side-by-side discussion of the two sets of findings. However, given that the three-way Group * Rehearsal Strategy * Plausibility interaction was not significant, we think that extensive discussion of the overlapping influences of rehearsal and schematic congruency on sleep-associated memory processing is unwarranted.

Page 18: *Memory retention at baseline was generally poorer for implausible than plausible noun-colour pairings, suggesting that the encoding of implausible pairings was relatively weak. Because previous work has shown that the memory benefits of sleep are more robust for weakly relative to strongly encoded materials (Denis et al., 2021, 2020; Payne, Chambers, & Kensinger, 2012; Schmidt et al., 2006, but also see Petzka et al., 2021), amplified overnight memory gains for implausible (vs plausible) pairings might reflect an impact of encoding strength, rather than schematic congruency, on sleep-associated consolidation. Indeed, as mentioned earlier, the seemingly delayed benefit of sleep for restudied information might have resulted from overall performance levels being generally high at the 12 h follow-up. Yet, if it were the case that implausible pairings were selectively strengthened during sleep because they were weakly encoded, then a three-way interaction should have emerged in our Group * Rehearsal Strategy * Plausibility ANOVA, with the weakest memories (i.e. non-rehearsed and implausible noun-colour pairings) showing the greatest enhancement across overnight sleep, but this was not the case.*

9) The authors argue that the results suggest restudy benefits sleep only if the sleep period comes soon after encoding. However, these effects could also be due to time of day benefits or interference over the wake-filled delay. If there is enough variability in the reported onset of sleep time, the authors could attempt to quantify the effect, seeing if perhaps a median split of the sleep group shows greater restudy benefits if sleep onset is closer to the end of the restudy session. However, I realize this may not be feasible if there isn’t enough variability/power.

**Authors:** Although this is an interesting point, we did not collect information on participants’ sleep onset time (only self-reported sleep duration was obtained), and thus cannot perform the subsidiary analyses suggested by the Reviewer.

We have, however, added the Reviewer’s suggestion to the Discussion as an avenue for future research:

Page 16: *Future online studies can assess this possibility more directly by asking participants to report their bed times and determining whether sleep-associated memory gains for restudied information are amplified among individuals who go to bed after a short (vs long) post-rehearsal delay. This could have potentially important practical implications for learning and education, such as the optimal timing of study sessions when students are preparing for examinations.*

10) The authors do not report if there was a significant Rehearsal Strategy x Plausibility interaction, separate from group (page 12). I assume none of the statistics from that analysis were significant, but including mention of the other findings from that ANOVA would be beneficial.

**Authors:** We thank the Reviewer for highlighting this missing information, which we have now added to our manuscript:

Page 14: *A significant Rehearsal Strategy * Plausibility interaction was observed at the 12 h follow-up F(2, 116) = 0.31, p = .029, η_p_^2^ = 0.06): the retention advantage for plausible (vs implausible) pairings was stronger in the no-rehearsal condition (t(59) = 7.60, p < .001, d = 0.98) than the retrieval practice (t(59) = 6.30, p < .001, d = 0.81) or restudy conditions (t(59) = 3.83, p < .001, d = 0.50). This interaction was not present at the 24 h follow-up (p > .05).*

11) Though the authors note that the order of the restudy and retrieval practice was counterbalanced across subjects, it would be interesting to know if there were any interesting carry over effects in this subject sample (e.g., restudy first leads to greater benefits vs. retrieval first).

**Authors:** The question of whether restudy (vs retrieval practice) first leads to greater sleep-associated memory benefits is an interesting one. However, we think that this exploratory analysis is unfeasible in our dataset because the small sample size (n=15 per counterbalancing condition) would make it highly underpowered. We have therefore opted to not carry out this exploratory analysis.

12) Missing citations in introduction: in the first paragraph, citations are needed for the line stating: ‘in the short term restudied information is better remembered than information subjected to retrieval practice’.

**Authors:** We thank the Reviewer for highlighting these missing citations, which we have now added to the Introduction:

Page 3: *In the short term (e.g. within an hour of learning), restudied information is better remembered than information subjected to retrieval practice (Bai, Bridger, & Zimmer, 2015; Kornell, Bjork, & Garcia, 2011; Roediger & Karpicke, 2006).*

13) Typo page 13, second sentence of discussion (" participants in the wake group had themselves had...")

**Authors:** We thank the Reviewer for highlighting this typo, which we have now corrected.

Page 15: *In keeping with the previously reported benefits of sleep for offline memory processing, source memory retention was higher at the 12 h follow-up in the sleep group than the wake group (an advantage that had subsided by the 24 h follow-up once participants in the wake group had slept).*
