# Supplementary material for: Sleep bolsters schematically incongruent memories
Source: PLoS One. 2022 Jun 24;17(6):e0269439. doi: 10.1371/journal.pone.0269439 (PMC9231735; doi:10.1371/journal.pone.0269439)
Supplement: Attachment — Submitted filename: PLOS_R2_ReviewerResponse_Final.docx [file pone.0269439.s003.docx]

**Response to Reviewers**

Reviewer #2: Overall, I appreciate the work the authors have put into this revision and the authors have sufficiently responded to most of my initial comments. However, I do have a few remaining questions.

1. In response to my question on the first set of reviews (#5) the authors noted the analysis looking at retention between 12 and 24 hour memory tests (e.g., 24 hr hits as proportion of 12 hr hits) did not yield a significant rehearsal strategy main effect nor a group x strategy interaction. However, this seems to counter the authors main claim is that sleep selectively benefits restudied items, and that this benefit may only be evident on the 24 hr test once potential ceiling effects decay. If so, wouldn’t the wake group, and/or the retrieval for the sleep group, be expected to show a decrease in accuracy with forgetting over time? It is possible that the lack of significant effects between the 12 to 24 hour memory is indicative the initial sleep vs. wake period following learning is critical for then what is fairly stable memory, but it’s difficult to reconcile this with the emergence of the interaction only at 24 hours – what then is driving the effect? I think the authors should mention the results of this analysis explicitly, and address how it can be interpreted in the context of the other results.

Authors: We thank the Reviewer for this additional comment. We are happy to report the full results of this exploratory analysis and have updated our Results section as follows (page 14):

*There was no main effect of Rehearsal Strategy (F(2,116) = 0.14, p = 0.87), no Group * Rehearsal Strategy interaction (F(2,116) = 0.69, p = .505) and no Rehearsal Strategy * Plausibility interaction (F(2,116) = 1.16, p = .318) in this exploratory analysis.*

The Reviewer has also pointed out that these null results are difficult to reconcile with our suggestion that the selective benefits of sleep on restudied information might emerge after the decay of ceiling effects, particularly given that the wake group exhibited an improvement in source memory recall between the 12 h and 24 h follow-ups. We thank the Reviewer for highlighting this point and have removed the discussion of potential ceiling effects from our manuscript.

The Reviewer has also asked us to address how the lack of a Group * Rehearsal Strategy interaction in this exploratory analysis of source memory retention between the 12 h and 24 h follow-ups can be reconciled with our planned analysis of the 24 h data (i.e. where retention was calculated relative to the baseline test and a significant Group * Rehearsal Strategy interaction emerged). We think that this is a good suggestion and have added the following text to our Discussion (page 16):

*If the benefits of sleep for restudied information are contingent on sleep taking place soon after learning, then one might have expected to observe a selective effect of sleep on restudied noun-colour pairings at both the 12 h and 24 h follow-ups. It is worth noting, however, that although a significant Group * Rehearsal Strategy interaction did not emerge at the 12 h follow-up, the pattern of results was very similar to that of the 24 h follow-up (where a significant Group * Rehearsal Strategy interaction did occur), with sleep providing the strongest benefit for restudied noun-colour pairings (see Figures 2 and 3). Interestingly, our exploratory analysis of the 24 h follow-up (with retention calculated relative to the 12 h follow-up) showed a main effect of Group, indicating that the wake group (who had slept between the 12 h and 24 h follow-ups) outperformed the sleep group (who had remained awake across the same interval). Yet, this analysis did not reveal a significant Group * Rehearsal Strategy interaction, suggesting that the memory benefits of delayed sleep are unaffected by prior re-engagement with the learned materials.*

2. For the Group x Rehearsal interaction at the 24 hour test: the authors note that the only the sleep group shows a restudy > retrieval effect. Does the sleep group (and/or the wake group) also show a benefit over the no rehearsal items, or is it specific to the restudy vs retrieval? If the wake group still shows a general benefit over the no rehearsal items, it still suggests any rehearsal conveys some benefit, but would speak further to the specific interaction of restudy and sleep. Please report the full results.

Authors: In both groups, retention scores were significantly higher in the restudy vs no-rehearsal condition and the retrieval practice vs no-rehearsal condition at the 24 h follow-up. This is clearly illustrated in Figure 3. Given that these effects are not driving the interaction in question, we would prefer to not include them in the main text because we think it will disrupt the readability and flow of our manuscript.

3. Item memory: The authors note the overall sleep vs wake effects for item memory, but do not analyze if there are any effects of rehearsal strategy or plausibility on item memory. It could be compelling if the authors could test if the reported effects are limited to associative source memory measures and are not seen for item memory itself. However, as this would be a full additional set of analyses, I leave it up to the authors’ discretion to include this suggestion.

Authors: We thank the Reviewer for this suggestion. However, because the rehearsal strategy and plausibility conditions do not have their own independent foils (a requirement for calculating our measure of recognition accuracy: d-prime), we cannot carry out this analysis.
